# Global alteration of colonic microRNAome landscape associated with inflammatory bowel disease

**DOI:** 10.3389/fimmu.2022.991346

**Published:** 2022-09-13

**Authors:** Éva Boros, Zoltán Hegedűs, Zoltán Kellermayer, Péter Balogh, István Nagy

**Affiliations:** ^1^ Seqomics Biotechnology Ltd., Mórahalom, Hungary; ^2^ Institute of Biochemistry, Biological Research Centre, Eötvös Loránd Research Network, Szeged, Hungary; ^3^ Institute of Biophysics, Biological Research Centre, Eötvös Loránd Research Network, Szeged, Hungary; ^4^ Department of Biochemistry and Medical Chemistry, Medical School, University of Pécs, Pécs, Hungary; ^5^ Department of Immunology and Biotechnology, University of Pécs, Pécs, Hungary; ^6^ Lymphoid Organogenesis Research Group, Szentágothai János Research Center, University of Pécs, Pécs, Hungary

**Keywords:** Crohn’s disease (CD), ulcerative colitis (UC), inflammatory bowel disease (IBD), non-coding RNAs, microRNA-seq

## Abstract

Inflammatory Bowel Disease (IBD) is characterized by chronic inflammation of the gastrointestinal tract that associates with, among others, increased risk of colorectal cancer. There is a growing evidence that miRNAs have important roles in pathological processes, such as inflammation or carcinogenesis. Understanding the molecular mechanisms such as alterations in microRNAome upon chronic intestinal inflammation is critical for understanding the exact pathomechanism of IBD. Hence, we conducted a genome wide microRNAome analysis by applying miRNA-Seq in a rat model of experimental colitis, validated the data by QPCR, examined the expression of a selection of precursor and mature miRNAs, performed in depth biological interpretation using Ingenuity Pathway Analysis and tested the obtained results on samples derived from human patients. We identified specific, interdependent expression pattern of activator/repressor transcription factors, miRNAs and their direct targets in the inflamed colon samples. Particularly, decreased expression of the miR-200 family members (miR-200a/b/c,-141, and -429) and miR-27b correlates with the reduced level of their enhancers (HNF1B, E2F1), elevated expression of their repressors (ZEB2, NFKB1) and increased expression of their target genes (ZEB2, RUNX1). Moreover, the marked upregulation of six miR-27b target genes (IFI16, GCA, CYP1B1, RUNX1, MEF2C and MMP13) in the inflamed colon tissues is a possible direct consequence of the lack of repression due to the downregulated miRNA-27b expression. Our data indicate that changes in microRNAome are associated with the pathophysiology of IBD, consequently, microRNAs offer potential targets for the diagnosis, prognosis and treatment of IBD.

## Introduction

Inflammatory Bowel Disease (IBD) is a generic term for chronic inflammatory conditions of the gastrointestinal (GI) tract. IBD symptoms, such as abdominal pain, malabsorption, bloating, diarrhea, bloody stool and fatigue considerably reduce the quality of patients life (1). The two main types of IBD are Crohn’s Disease (CD) and Ulcerative Colitis (UC) with broadly similar pathogenesis, except the location of the involved sections along the GI tract and the depth of ulcers through the tissue wall (2). Transmural patchy lesions from the mouth to the rectum are characteristics of CD while UC is limited to the upper layer of colon tissue (3, 4). As a multifactorial disorder, genetic, microbial and environmental factors play a role in the development of IBD but the exact molecular background and pathogenesis is still unknown (5–7). The prevalence of IBD is increasing worldwide emphasizing the importance of novel diagnostic and therapeutic strategies (8).

The knowledge on non-coding RNAs (ncRNAs) is rapidly growing including their function(s) in different pathological conditions, among others IBD (9). microRNAs (miRNAs) belong to ncRNAs and have an important role in the fine-tuning of gene expression. These small, 18-21 nucleotide long single-strand RNA molecules are able to bind the 3’UTR region of their target messenger RNAs (mRNAs), thereby either inhibiting their translation into proteins or inducing mRNA degradation. miRNAs are transcribed from coding or non-coding regions of the genome as primary precursor miRNA (pri-miRNA) transcripts. These precursors are translocated from the nucleus to the cytosol and cleaved by several enzymes to get the mature, functional miRNA form (10). miRNA expression is regulated by transcription factors, by methylation at transcriptional level as well as through miRNA processing or miRNA stability mechanisms at post-transcriptional level (11).

The history of miRNAs in human disorders has begun with the discovery of their function in carcinogenesis (12). Ever since, many studies reported the correlation between dysregulated miRNA expression and human disorders e.g. cardiovascular (13, 14), neurodevelopmental (15, 16) or inflammatory diseases (17, 18). Until now, the role of miRNAs in IBD was mostly examined in the context of IBD associated genes and pathways (19–21). We have recently highlighted the relevance of miRNAs upon epithelial-to-mesenchymal transition (EMT) underlying IBD (22). During EMT, epithelial cells disintegrate cell-cell connections and became motile, invasive mesenchymal-like cells, a phenomenon that leads to metastasis and tumorigenesis (23). We have reported the association between downregulation of selected miRNAs and upregulation of their target genes in the inflamed colon tissues that may drive EMT and contribute to the development of IBD-related colorectal cancer (CRC) (22).

In this work, we performed miRNA-Seq analysis of an *in vivo* rat model of IBD that we interpreted together with our previous whole transcriptome analysis data (24) to get insight into the complex regulatory network of transcriptional factors, miRNAs and their target genes. As model, we used the chemical irritant 2,4,6-trinitrobenzene sulfonic acid (TNBS) as a well-established rat model of IBD induction, allowing the analysis of various pathogenic factors (25). In addition, we examined these regulatory circuits on samples derived from colon biopsies of IBD patients and *in vitro* cell cultures in order to prove the relevance of the data obtained on rat samples. The presented data show a specific, interdependent expression pattern of activator/repressor transcription factors, miRNAs as well as their direct targets that may have the potential to boost not just EMT but chronic inflammation in the colon of IBD patients.

## Materials and methods

### *In vivo* rat model and sample collection

All procedures were performed in accordance with the standards of the European Community guidelines for the Care and Use of Laboratory Animals and were approved by the Institutional Ethics Committee at the University of Szeged (XX./4799/2015). To investigate the molecular background of IBD we used the 2,4,6-trinitrobenzene sulfonic acid (TNBS) induced rat model of experimental colitis as described previously (26). Briefly, male Wistar rats (180–220 g) were randomly divided into two groups: the first group served as control and the second group was treated with TNBS (colitis-induced) based on the method described by Morris et al. (27). Following overnight fasting, animals were challenged with a single dose of 0.25 mL TNBS (10mg in 0.25ml of 50% ethanol, w/v) intracolonically with an 8 cm long soft polyethylene tube through the anus under mild anesthesia (thiopental, i.p. 40 mg/kg). In the case of the control group (n=2), samples were taken from random colon sections; samples from colitis-induced animals were taken from inflamed colon region (n=4) as well as from non-adjacent uninflamed region (n=4). The damage score and lesion measurement (extents of macroscopically apparent inflammation, ulceration, and tissue necrosis) were determined using computerized planimetry as described previously (28). All samples were kept in TRIzol reagent (Thermo Fisher) at -80°C.

### IBD patients

Colonic biopsies were obtained from 21 consenting patients with IBD [13 females and 8 males; median age 41 years, 25-74 years (for more details see (24)] undergoing colonoscopy for diagnostic purposes approved by Hungarian Medical Research Council (ETT) Medical Research Council’s Committee of Scientific and Research Ethics (TUKEB) (license number 59-32-PTE2015/IBD) as described previously (29). Human colonic biopsies were obtained in accordance with the guidelines set out by the Medical Research Council of Hungary. Sample collection and classification was performed according to disease status of patients, active/relapsing or inactive/remission phase. Furthermore, samples from relapsing patients were subdivided as uninflamed or inflamed according to the status of colon tissue. To classify uninflamed from inflamed samples, macroscopic diagnostic features were used in accordance with the recommendations of the European Crohn’s and Colitis Organisation (30). Multiple samples were obtained both from visible lesions and from mucosa which is normal on gross inspection. Classically, the mucosa in UC has a friable granular appearance and shows superficial ulcers. The earliest visible mucosal lesions of CD are small aphthous ulcers after which they coalesce to large deep serpiginous or linear ulcers (24).

### Cell culture conditions and stimulations

The human THP-1 monocytic cells and HT-29 colonic epithelial cells were maintained in RPMI-1640 (Gibco) or DMEM (Gibco) medium, respectively, supplemented with 10% fetal bovine serum (FBS; Gibco) and 1% antibiotic/antimycotic solution containing 10,000 units/ml of penicillin, 10,000 µg/ml of streptomycin and 25 µg/ml of Amphotericin B (Gibco). Cells were cultured at 37°C in an atmosphere of 5% (v/v) CO_2_ in air. For TNFα or LPS treatment, cells were seeded into six-well cell culture plates (Sarstedt) and stimulated with 1 μg/ml LPS (Sigma) or 10 ng/ml TNFα (R&D Systems) for the indicated time, followed by RNA extraction.

### Extraction of total RNA

Samples from rat colons were homogenized in TRIzol reagent by ULTRA-TURRAX T-18 (IKA) instrument as described previously (26). 0.1 ml of chloroform (Sigma) was added to 0.3 ml homogenized sample with vigorous vortexing. Samples were centrifuged at 13000 rpm for 10 minutes. Total RNA was than extracted from the upper aqueous phase. RNeasy Plus Mini Kit (Qiagen) was used to purify total RNA from rat colon samples, as well as human THP-1 and HT-29 cells according to the manufacturer’s protocol. Total RNA from human biopsies was isolated using NucleoSpin RNA Kit (Macherey-Nagel) according to the manufacturer’s protocol. The quality and the quantity of the extracted RNAs were determined by TapeStation (Agilent) and Qubit Fluorometer (Thermo Fisher).

### Reverse transcription and quantitative real-time PCR (QPCR)

Reverse transcription was performed by SuperScript VILO Master Mix (Thermo Fisher) according to the manufacturer’s instructions. Gene and microRNA expression was measured by quantitative real-time PCR using the StepOne PCR Systems (Thermo Fisher). SybrGreen technology based QPCR reactions were performed by SYBR Select Master Mix (Thermo Fisher) with specific exon spanning primer sets (**Table 1**); TaqMan technology based reactions were performed by TaqMan Fast Advanced Master Mix and TaqMan Gene Expression Assays (Thermo Fisher) (**Table 2**). The ratio of each gene relative to the 18S rRNA was calculated using the 2^-ΔΔCT^ method.

**Table 1:** SybrGreen primer sets used in QPCR experiments.

Gene ID	Forward (5’- 3’)	Reverse (5’- 3’)
PRI-MIR-27B	TCACATTGCCAGGGATTACCA	AGCTAAGCTCTGCACCTTGTT
PRE-MIR-27B	CCTCTCTAACAAGGTGCAGAGC	GCAGAACTTAGCCACTGTGAAC
PRE-MIR-223	CGCTCCGTGTATTTGACAAGC	TGACAAACTGACACTCTACCACA
E2F1	CTTGAGGGCATCCAGCTCATT	GCAATGCTACGAAGGTCCTGA
MMP13	AGGAGCATGGCGACTTCTAC	AGACCTAAGGAGTGGCCGAA
RUNX1	CCTCAGGTTTGTCGGTCGAA	CTGCCGATGTCTTCGAGGTT
MEF2C	CACAGGTGGTCTGATGGGTG	CCTGGTGAGTTTCGGGGATT
CYP1B1	AACGTACCGGCCACTATCAC	TCACCCATACAAGGCAGACG

**Table 2:** TaqMan assays used in QPCR experiments. Note that due to high conservation, the miRNA assays are both applicable for rat and human miRNAs.

Gene ID	Assay number
miR-200a-3p	tm000502
miR-200b-3p	tm002251
miR-200c-3p	tm002300
miR-141-3p	tm000463
miR-429-3p	tm001077
miR-27b-3p	tm000409
miR-223-3p	tm000526
miR-31-5p	tm002279
RNU48	tm001006
U6	tm001973
18S	hs99999901_s1

For miRNA detection TaqMan MicroRNA Reverse Transcription Kit (Thermo Fisher) and TaqMan Universal PCR Master Mix (Thermo Fisher) were used according to the manufacturer’s instructions. The specific miRNA assays were purchased from Thermo Fisher; assay numbers are shown in **Table 2**. The ratio of each miRNA relative to the endogenous U6 or RNU48 snRNA for rat or human, respectively, was calculated using the 2^-ΔΔCT^ method.

### miRNA sequencing and bioinformatic analysis

miRNA-Seq was performed by TruSeq Small RNA Library Preparation Kit (Illumina), according to the manufacturer’s instructions and sequenced on Illumina MiSeq instrument. Briefly, during 3’ adapter ligation reactions achieved by the T4 RNA Ligase 2 (Deletion Mutant) enzyme, 1 μg total RNA was used from each rat colon sample. After 5’ adapter ligation by T4 RNA Ligase, reverse transcription was performed by SuperScript II Reverse Transcriptase (Thermo Fisher), and the constructs were amplified by PCR. Amplification products were verified on TapeStation High Sense DNA tape (Agilent). cDNA contracts were purified and size selected by gel electrophoresis on 6% Novex TBE gels (Thermo Fisher). To elute the DNA, gel slices were broken and incubated in elution buffer overnight. Gel slurry was removed by Ambion Spin Column, and the DNA was precipitated with isopropanol. Resuspended DNA libraries were quality checked on TapeStation High Sense DNA tape. Final libraries were quantified by Qubit (Thermo Fisher) and sequenced on Illumina MiSeq instrument using the 50-base sequencing chemistry.

The quality checking and filtering of raw Illumina reads was carried out by the QC for Sequencing Reads, Trim Reads modules of CLCBio Genomic Workbench v11.0.1 (Qiagen). The bioinformatics and statistical analysis of the miRNA expression was done by the Small RNA Analysis, Annotate and Merge Counts, Principal Component Analysis and the Empirical Analysis of DGE modules of the same software. Sequenced reads were annotated and counted according the mature *Rattus norvegicus* miRNA sequence records of the miRBase Database (Release 22.1). The differential expression of miRNAs was considered as significant when the abs(fold change) > 2x and the FDR < 0.05 selection criteria were fulfilled. The downstream gene network studies were performed by Ingenuity Pathway Analysis (IPA, Qiagen) software. Hierarchical clustering and the generation of the heatmap representation of miRNA expression were done by the heatmap.2 function of gplot R package.

### Statistical analysis and data representation

Statistical evaluations were performed using the IBM SPSS Statistics program for Windows. Graphs were plotted with GraphPad Prism 6 software. Quantitative data are presented as the mean ± SEM and the significance of difference between sets of data was determined by one-way analysis of variance (ANOVA) following LSD *post-hoc*t test; a *p* value of less than 0.05 was considered significant.

## Results

### TNBS-induced colitis alters miRNA expression profiles

We started by exploring the extent of global pri-miRNA changes in a rat *in vivo* model of experimental colitis. To this end, we analyzed RNA-Seq data of the well-characterized 2,4,6-trinitrobenzene sulphonic acid (TNBS) induced rat model of colitis, in which the inflammatory response is due to the generation of transmural oxidative stress and release of proinflammatory mediators. Specifically, in our previous study, we reported that the whole transcriptome analysis of the TNBS induced rat model of IBD revealed significant differences between the inflamed and uninflamed colon samples of TNBS treated rats that associated with the activation of inflammation and carcinogenesis related canonical signaling pathways (24). Here, we again used this RNA-Seq dataset (GEO accession GSE149517) and focused on the expression of pri-miRNAs, the precursors of miRNAs.

**Figure 1:**
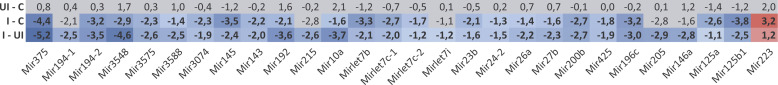
Global downregulation was characteristic for the expression of miRNA precursors in the involved colon samples of TNBS induced rat model of IBD as determined by RNA-Seq. Heat map shows expression alteration of miRNA precursors between control, TNBS treated uninflamed and TNBS treated inflamed rat colon samples. Values are given in logarithmic scale; red indicates at least 2-fold significant increase (≥ log 1; *p* < 0.05), blue indicates at least 2-fold significant decrease (≤ log -1; *p* < 0.05), gray labels non-significant change (*p* ≥ 0.05) in expression level. Abbreviations as “UI – C”, “I - C” and “I – UI” represent the TNBS treated – uninflamed (UI) vs. control **(C)**, TNBS-treated – inflamed **(I)** vs. control **(C)** and TNBS treated - inflamed **(I)** vs. TNBS treated – uninflamed (UI) rat colon sample comparisons.

The number of known microRNAs is continuously increasing, the number of annotated human and rat precursor/mature miRNA at the time this study was conducted was 1917/2564 and 496/764, respectively, according to the reference repository miRBase release 22 (31). In the TNBS treated rat colons, we identified 28 miRNA precursors with significantly altered expression in the inflamed tissues (**Figure 1**). Notably, 27 out of 28 pri-miRNAs (eg miR-192, -200b, -375) showed a downregulated expression profile in the inflamed rat colon samples, with only miR-223 exhibiting an upregulated expression (**Figure 1**).

**Figure 2:**
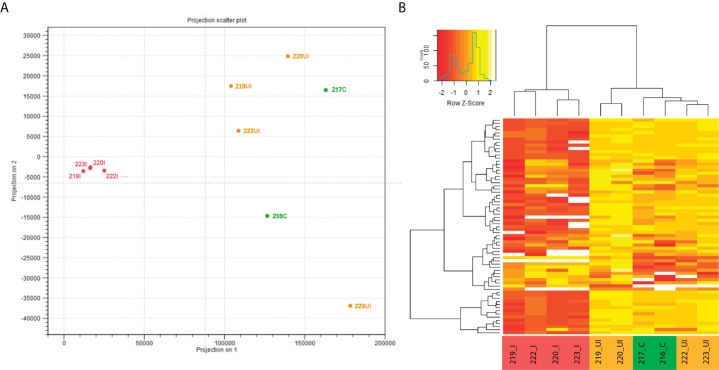
*In silico* performed PCA of individual miRNA expression data clustered samples into control, TNBS-treated uninflamed and TNBS-treated inflamed groups, while examination of significantly altered miRNAs confirmed these observations. **(A)** Principal component analysis (PCA) of individual rat colon samples and **(B)** representative heat map image of significantly altered miRNAs in the control (green, n=2), TNBS-treated uninflamed (orange, n=4), TNBS-treated inflamed (red, n=4) tissues. Heat map contains 68 miRNAs that displayed significant expression change (white representing missing values). Abbreviations “C”, “UI” and “I” in sample IDs represent control, uninflamed and inflamed sample groups, respectively.

Next, we applied miRNA-Seq approach for two reasons. First, non-coding RNAs, including miRNAs and their precursors, form a very small portion of the total RNA content of a mammalian cell (32), hence their detection and quantification by RNA-Seq highly depends on the sequencing depth and abundance of the pri-miRNA. Second, pri-miRNAs are processed, first into pre-miRNAs and subsequently to mature miRNAs, which are known regulators of protein expression. In light of these, and in order to monitor the global, unbiased expression profile of functional miRNAs, miRNA-Seq was conducted from the same input samples we used previously for transcriptome (RNA-Seq) profiling. Next, we performed *in silico* principal component analysis (PCA) of individual samples to visually summarize normalized miRNA expression profiles revealing similarities and differences between samples. We determined that TNBS-treated inflamed rat colon samples (n=4) form a close group that clearly clusters apart from TNBS-treated uninflamed (n=4) as well as control (n=2) samples (**Figure 2A**). In addition, heat map representation of significantly altered miRNAs confirmed differences between inflamed and uninflamed samples of TNBS treated animals, whereas control and uninflamed samples proved to be very similar at miRNA expression level (**Figure 2B**). These data are in perfect agreement with those previously reported at the global transcriptome level (24).

We report that 9% (68) of the 764 rat miRNAs are significantly up- or downregulated in inflamed samples (i.e. show at least a two-fold change with an FDR-adjusted *p*-value < 0.05; **Figure 2B** and **Supplementary Table 1**). Interestingly, nearly 65% (44/68) of the miRNAs with altered expression show decrease in the inflamed colon regions. General down-regulation of mature miRNAs as determined by miRNA-Seq (**Figure 2**) is in perfect agreement with down-regulation of pri-miRNAs observed in RNA-Seq experimental setup (**Figure 1**).

### Correlation between precursor and mature miRNA expression

We previously reported decreased expression of miR-375, miR-192, miR-200b, miR-30a and miR-199a in the inflamed colon regions of TNBS-treated rats and in IBD patients (22, 26, 29). Here we sought to validate the newly established miRNA-Seq dataset by monitoring the expression of these five miRNAs. We found a decreased expression pattern (**Supplementary Table 1**) of all five miRNAs (miRNA-Seq) that perfectly resembled the previously reported decreased expression pattern these miRNAs (QPCR), indicating that the miRNA-Seq data are of high quality, thus allowing further analysis.

We next sought to assess the extent of the association of RNA-Seq and miRNA-Seq data with respect to pri-miRNA and mature miRNA expression. To this end, we examined the correlation between the previously performed transcriptome analysis (RNA-Seq; **Figure 1**) and miRNA-Seq (**Supplementary Table 1**) data, resembling pri-miRNA and mature miRNA expression, respectively. We determined that the expression change of numerous miRNAs was detected at both precursor and mature miRNA level (eg. miR-375, miR-194, miR-192, miR-215, miR-26a, miR-27b, miR-200b, miR-196c, miR-205 and miR-223). These data show that the expression pattern of mature miRNAs perfectly follows that of the precursors. It is important to notice, however, that miRNA-Seq detected more significantly changed mature microRNAs. This may be due to 1) the fact that several mature miRNAs are originating from the very same precursor (eg. the miRBase contains 496 precursor and 764 mature rat miRNAs) and 2) the limitations of the RNA-Seq technology which seemingly detected only the most abundant microRNA precursors.

### Dysregulated miRNA expression in inflamed colon may lead to cancer formation

For the genome-wide (RNA-Seq and miRNA-Seq) examination of inflammatory processes in the bowel, we used the 2,4,6-trinitrobenzene sulphonic acid (TNBS) induced rat *in vivo* model of experimental colitis. Here we aimed to validate the findings made using the murine model on colon biopsies derived from IBD patients. Both the chemically triggered rat tissues and the colons of IBD patients are characterized by the alteration of inflamed and intact regions (24, 26, 29). In order to get a comprehensive view of processes in these tissues, we simultaneously examined the expression of miRNAs both in macroscopically normal (uninflamed) and in inflamed regions.

**Figure 3:**
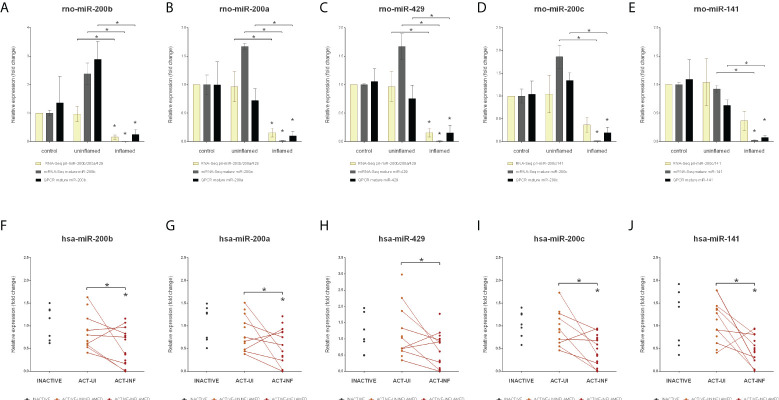
Decreased expression of the miR-200 family members in inflamed colons. Relative expressions of pri-miR-200b/200c/429 and mature miR-200b **(A)**, miR-200a **(B)**, miR-429 **(C)**, and pri-miR-200c/141 and mature miR-200c **(D)** and miR-141 **(E)** are shown from control (left columns), uninflamed (middle columns) and inflamed (right columns) rat colon sections; yellow, gray and black columns represent RNA-Seq (pri-miRNA), miRNA-Seq (mature miRNA) and QPCR (mature miRNA) data, respectively. Data are presented as the mean ± SEM; *p<0.05. In the bottom **(F–J)** panels expression changes of the same microRNAs are shown from inactive (left), active uninflamed (middle) and active inflamed (right) colon samples of IBD patients. Dots represent individual values, the lines connect uninflamed and inflamed samples from the same IBD patient; *p<0.05.

The expression of several miRNAs, including miR-200b known to play a role in the epithelial to mesenchymal transition (EMT), a fundamental process in wound healing as well as in formation of metastasis (33, 34), is downregulated in the model of in experimental colitis (26). MiR-200b, along with miR-200a, -200c, -141 and -429, is a member of the miR-200 family, which forms clusters in the genome. In human, cluster 1 consists of miR-200a/b and -429 and cluster 2 includes miR-200c and -141. To further investigate the possible dysregulation of the EMT in intestinal inflammation, we next examined the expression of the miR-200 family. We determined that the expression of the rat precursor and mature miRNAs belonging to the miR-200 family (miR-200a/b/c, -141 and -429) strongly decreased in the inflamed tissues (**Figure 1** and **Supplementary Table 1**). To further validate these data, we next measured the expression of mature miRNAs by QPCR and observed perfect overall correlation with our current miRNA-Seq results both in the inflamed colons of TNBS treated rats (**Figures 3A–E**) and, more importantly, in IBD patients (**Figures 3F–J**).

**Figure 4:**
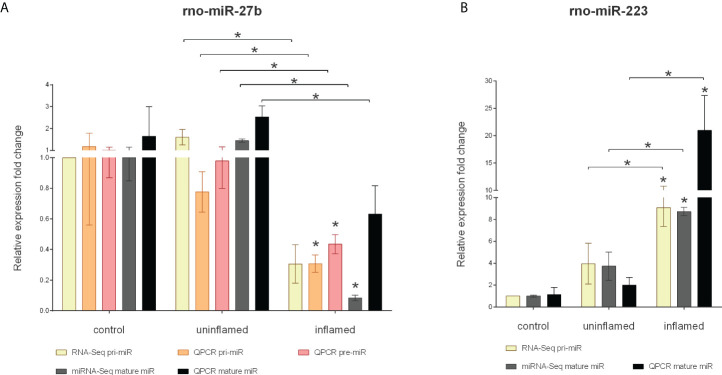
Expression changes of precursors and mature miR-27b and miR-223 in the inflamed colons of experimentally induced rat model of IBD. Relative expression of pri-, pre- and mature miR-27b **(A)** is shown from control (left columns), uninflamed (middle columns) and inflamed (right columns) rat colon sections; yellow and gray columns represent RNA-Seq and miRNA-Seq data, while orange, red and black present QPCR results. On panel **(B)**, relative expression of pri-and mature miR-223 is shown from control (left columns), uninflamed (middle columns) and inflamed (right columns) rat colon sections; yellow and gray columns represent RNA-Seq and miRNA-Seq data, while black presents QPCR results. Data are presented as the mean ± SEM; *p<0.05.

**Figure 5:**
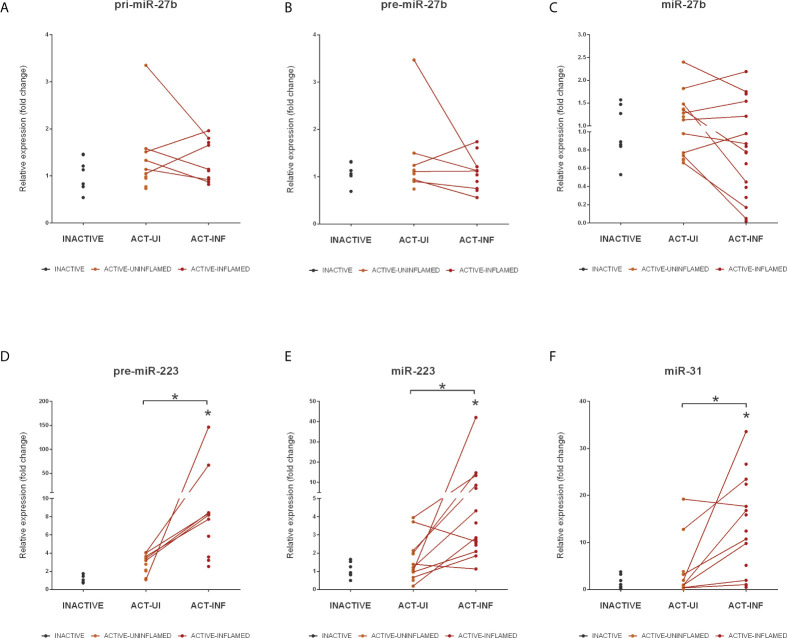
Expression changes of miR-27b, miR-223 and miR-31 in the inflamed colons of IBD patients. Relative expression of pri- **(A)**, pre- **(B)** and mature **(C)** miR-27b, pre- **(D)** and mature **(E)** miR-223 and mature miR-31 **(F)** is shown from inactive (grey), active uninflamed (orange) and active inflamed (red) colon samples of IBD patients. Dots represent individual values; data are presented as the mean ± SEM. Lines connect uninflamed and inflamed samples from the same IBD patient; *p<0.05.

MiRNA-Seq identified two other, highly conserved microRNAs, miR-223 and miR-27b, with significantly altered expression level (**Supplementary Table 1**). These miRNAs act as tumor promoting oncogenes or suppressors, the function being context- or tissue-dependent (35, 36). In colorectal cancer (CRC), the expression of miR-27b is downregulated while the level of miR-223 directly correlates with disease severity with higher expression associated with more severe symptoms (36, 37). Here we sought to assess the role of miRNA dysregulation in CRC formation, both at microRNA biogenesis and species (rat, human) level. To this end, we first measured the expression changes of pri-, pre- and mature miR-27b and identified a significant decrease at all levels in rat samples (**Figure 4A**) and a moderate decrease of human miR-27b (**Figures 5A–C**). In contrast, the expression of pri- and mature miR-223 markedly increased in both inflamed rat colons (**Figure 4B**) as well as active inflamed human samples (**Figures 5D, E**). This analysis confirmed that the alteration of miRNA expression occurs already at the precursor level and that this phenomenon takes place upon both rat and human colon inflammation.

In addition to miRNAs that significantly changed at precursor level, we also determined the expression pattern of miR-31. Similarly to miR-223 and -27b, miR-31 has a dual function in cancer progression: while in e.g. ovarian, prostate or gastric cancer it acts as tumor repressor, in CRC tissues its elevated expression promotes carcinogenesis (38). As revealed by miRNA-Seq, miR-31 was upregulated in the inflamed rat colon samples (**Supplementary Table 1**) that is in concordance with the elevated expression of miR-31 in the colon biopsies derived from IBD patients (**Figure 5F**).

Overall, these findings suggest that an important step in the development of IBD-related colorectal cancer is the dysregulated microRNA expression, which is present at all levels (including pri-, pre- and mature miRNA expression) of microRNA biogenesis.

### Interplay between transcription factors and miRNAs

Transcription factors (TFs) known to play a role in EMT (e.g. ZEB2, SNAI1) exhibit increased expression in the inflamed rat and human colon regions which, in turn, is associated with the downregulation of their inhibitory miRNAs (e.g. miR-200b, -192, -375, -143, -30a, -107, -199a and -34a) (22, 26, 29). To further investigate the parallel expression patterns of transcription factors and miRNAs in inflamed colon, we compared the transcriptome and miRNA expression profiles *in silico*. For this, we searched known transcriptional regulators of the significantly altered miRNAs using the TransmiR database (39, 40). We primarily focused on those miRNAs for which both the precursor and the mature miRNA exhibited an expression change (miR-200 family, miR-27b and miR-223) as we hypothesized that their altered expression may be directly associated with a given transcription factor. We narrowed the list to those transcription factors that have experimentally validated regulatory roles in miRNA expression; furthermore, listed only those that have altered expression in inflamed colon as determined by RNA-Seq (**Table 3**). Consistent with the hypothesis, we observed a clear correlation between expression change of TFs and their regulated miRNAs. Namely, decreased expression of enhancer (e.g. E2F1, HNF1B) and increased expression of repressor (e.g. NFKB1, ZEB2) TFs associated with the downregulation of miR-200 family members and miR-27b. In contrast, elevated miR-223 and miR-31 expression appears to be the consequence of the upregulation of activator (e.g. CEBPB) and decrease of repressor (e.g. E2F1) TFs in the inflamed colon.

**Table 3:** Expression changes of miRNAs and their regulator transcription factors in the inflamed colon.

	Transcription factor	Type of regulation	Regulated miRNA	Reference
**Downregulated transcription factors**	**HNF1B**	Activation	miR-200 family	(41, 42)
	**HNF4A**	Activation	miR-200 family	(43)
	**KLF4**	Activation	miR-200 family	(44)
	**KLF5**	Activation	miR-200 family	(45)
	**MYB**	Activation	miR-200 family	(46)
	**GRHL2**	Activation	miR-200 family	(47, 48)
	**E2F1**	Activation	miR-27b	(49)
	**E2F1**	Repression	miR-223	(50)
**Upregulated transcription factors**	**ZEB2**	Repression	miR-200 family	(34)
	**SNAI1**	Repression	miR-200 family	(51)
	**PELP1**	Repression	miR-200 family	(52)
	**TGFB1**	Repression	miR-200 family	(53)
	**NFKB1**	Repression	miR-200 family	(54)
	**NFKB1**	Repression	miR-27b	(55)
	**TWIST1**	Repression	miR-200 family	(56)
	**TWIST1**	Activation	miR-223	(57)
	**SPI1**	Activation	miR-223	(58)
	**NOTCH1**	Activation	miR-223	(59)
	**CEBPB**	Activation	miR-223	(58, 60)
	**CEBPB**	Activation	miR-31	(61)

Colour of the text indicates the direction of gene- or miRNA expression alteration, blue: decreased expression, red: increased expression of transcription factors or miRNAs. References indicate the experimentally validated regulatory effect of transcription factors on the given miRNA/s.

**Figure 6:**
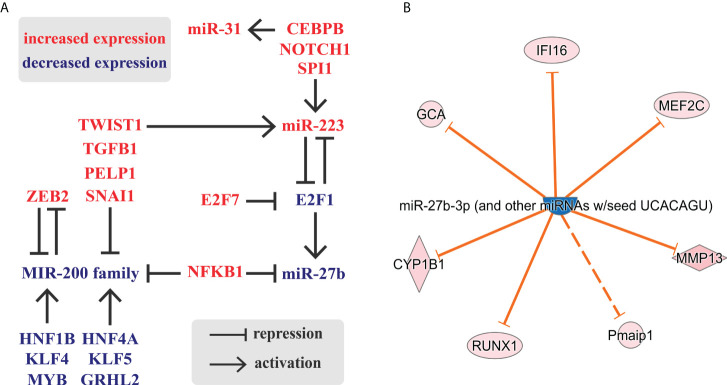
Schematic representation of a possible regulatory circuit of transcription factors and microRNAs during colon inflammation. **(A)** The comparison of inflamed to uninflamed colon samples identified genes and miRNAs (RNA-Seq (24) and miRNA-Seq (this study), respectively) with altered expression. **(B)** Selection of miR-27b targets with altered expression in the inflamed rat colon samples based on RNA-Seq data and IPA knowledge base. Legend of molecule shapes is available from reference (62).

The results above show an intimate relation of TF and miRNA expression in inflamed colon, in which numerous molecules with altered expression can be grouped into a single regulatory circuit (**Figure 6A**). Notably, we identified the miR-223-E2F1-miR-27b slice of this network as a possible central node that includes a feed-back loop, inhibitory and enhancing units. E2F transcription factors have a role in cell cycle regulation, differentiation and development. Canonical E2F protein, E2F1 is able to bind miR-27b promoter and induces miR-27b transcription (49). In contrast, E2F1 and miR-223 form a feed-back loop, in which E2F1 acts as a transcriptional repressor of miR-223 which, in turn, inhibits E2F1 translation (50). In the inflamed colon, upregulation of miR-223 and downregulation of E2F1 is consistent with the reduced expression of miR-27b (**Figure 6A**). Overall, this expression pattern indicates the potential role of miR-27b target genes in the regulation of colon inflammation.

### Increased expression of miR-27b targets in inflamed colon

To test this, we next applied Ingenuity Pathway Analysis (IPA) to identify mRNAs that may be affected by this regulatory circuit. We focused on miR-27b targets which exhibited altered expression, again limiting hits to the experimentally validated connections (**Figure 6B**). We identified seven miR-27b targets with altered expression, yet excluded Pmaip1 (Phorbol-12-myristate-13-acetate-induced protein 1, also known as NOXA) from further analysis because its interaction with miR-27b is indirect according to Ingenuity Knowledge Base.

IFI16 (interferon gamma inducible protein 16) is an intracellular DNA sensor that regulates interferon expression, thereby playing a role in innate immune responses (63). Its pathological expression has been reported from colons of IBD patients where, as a target of autoantibodies, it triggers the malfunction of immune homeostasis (64). Similarly, GCA (grancalcin) induces interferon production by the interaction with TLR9 (Toll-like Receptor 9); in addition, it plays a role in autophagy regulation (65, 66). CYP1B1 (cytochrome P450 family 1 subfamily B member 1) enzyme is a member of the cytochrome P450 (CYP450) family that plays a role in the conversion of procarcinogens to carcinogens, in addition, it is involved in the process of CRC progression (67). RUNX1 (Runt-related transcription factor 1) also acts as an oncogene in epithelial tumors; it induces CRC metastasis through the Wnt/β-catenin and EMT pathway activation (68). Transcription factor MEF2C (Myocyte Enhancer Factor-2 C) regulates B- and T-cell development, has an oncogenic function in acute myeloid leukemia and its upregulation is directly associated with CRC progression (69). Finally, MMP13 (matrix metallopeptidase 13) is a collagenase that takes part in the activation of inactive TNFα (tumor necrosis factor alpha), a crucial cytokine in IBD pathogenesis and therapy (70).

**Figure 7:**
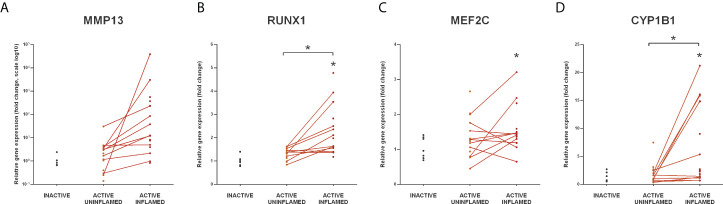
Increased expression of miR-27b targets in the inflamed human colon samples. Relative expression of MMP13 **(A)**, RUNX1 **(B)**, MEF2C **(C)** and CYP1B1 **(D)** is shown from inactive (left), active uninflamed (middle) and active inflamed (right) colon samples of IBD patients. Dots represent individual values, and the lines connect uninflamed and inflamed samples from the same IBD patient; *p<0.05.

Since all six genes were upregulated in the inflamed colon of TNBS induced rats (**Figure 6B**), we next examined the expression of CYP1B1, RUNX1, MEF2C and MMP13 (71–74) in the colon samples of IBD patients. We observed a marked upregulation in the inflamed colon tissues of all four genes (**Figure 7**), which may be a direct consequence of the lack of repression due to the downregulated miRNA-27b expression (**Figure 5C**).

### Dysregulated miR-223, miR-27b, E2F1 and MM13 expression may originate from different cell types

Finally, we asked whether the global expression changes we detected in colon tissues indeed originate from different cell types. Importantly, miR-223 is the predominant cargo of microvesicles and exosomes in peripheral blood and neutrophil-derived extracellular vesicles; furthermore, polymorphonuclear leukocytes are able to transfer miR-223 containing cargo to alveolar-epithelial cells (35). Therefore, we hypothesized that some of the genes and miRNAs with altered expression in inflamed colon tissues are cell type specific alterations of molecules which exhibit their effects on a different cell type.

**Figure 8:**
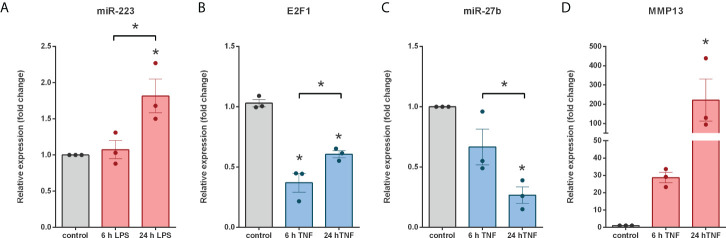
Coherent expression changes of miRNAs, miRNA targets and regulating transcription factors in LPS treated THP-1 cells and TNFα triggered HT-29 colonic epithelial cells. Relative expression of miR-223 **(A)** in LPS treated THP-1 cells, and E2F1 **(B)**, miR-27b **(C)** and MMP13 **(D)** in TNFα activated HT-29 cells. Data are presented as the mean ± SEM (n=3);*p<0.05.

To test this hypothesis, we examined the miR-223-E2F1-miR-27b regulatory node - for which direct relationship between the molecules has been previously demonstrated (49, 50, 74) - by monitoring the expression pattern of the members using *in vitro* cell cultures. Since Caco-2, HT29 and THP-1 cell are well known human-derived *in vitro* models of IBD (75) we used these cell lines in our further experiments. miRNAs often have cell type specific manifestation, such as the expression pattern of miR-223 which is primarily expressed by myeloid cells (76, 77). Importantly, the initial source of miR-223 in inflamed colon tissue of DSS-induced colitis is from infiltrating neutrophils and monocytes (78). In line with this, we could not detect miR-223 expression either in untreated or in TNFα activated HT-29 colonic epithelial cells. Hence, we used THP-1 leukemia monocytic cells and stimulated them with lipopolysaccharide (LPS) to monitor the inducibility of miR-223. While 6 h of LPS induction had no effect on miR-223 expression, 24 h of LPS stimulation significantly increased its expression (**Figure 8A**). Since we could not detect miR-27b expression neither in unstimulated nor in LPS stimulated Caco-2 cells (data not shown), we next activated HT-29 colonic epithelial cells with TNFα to mimic an inflammatory environment. We detected significantly decreased expression of both miR-27b and its transcriptional activator E2F1 (**Figures 8 B, C**). As a consequence of the possible deregulation, the mRNA level of the miR-27b target MMP13 was markedly upregulated (**Figure 8D**).

Collectively, these results suggest that while cell-type specific differences in miRNA pattern exist between various gut components both in human patients and chemically induced colitis in rat, cellular or soluble cytokine-mediated interactions during inflammation may further modulate the miRNA expression profiles in IBD.

## Discussion

According to Kaplan and Windsor, the global evolution of IBD stepped into the stage of “Compounding Prevalence” in the countries of the Western world and may reach “Prevalence Equilibrium” stage in 30 years (79). In industrialized countries of the Western world IBD prevalence increased from 0.5% to 0.75% between 2010 to 2020 and is projected to reach 1% by 2030 (79). Although the incidence range of IBD is stabilizing in the Western countries (12-26 per 100,000 person), new cases raise the prevalence because of the low mortality (79). These trends highlight the importance of exploring novel targets and therapeutic strategies to support the health-care system in tackling the upcoming burden.

MicroRNAs are considered central regulators of gene expression, and miRNA expression may be predictive of underlying molecular mechanisms in complex diseases such as IBD. Dextran sodium sulfate (DSS)- and TNBS-induced colitis are widely used as rodent IBD models (80). Inflammation in DSS seems to be caused by hyperosmotic damage and is confined to the mucosa and lamina propria, in contrast to the TNBS-model where inflammation is hapten-mediated and transmural, therefore the pathological tissue lesions are more closely reminiscent to the human conditions. Hence, we used the rat model of experimental colitis and analyzed the biogenesis and expression pattern of miRNAs, comparing it to gene expression alterations. As miRNAs exhibit variable expression between species and distinct cell types, we also used samples from IBD patients and human cells, in order to provide greater significance to our findings.

By using an integrated analysis of RNA-Seq and microRNA-Seq we depicted miRNA-mRNA networks involved in IBD formation/maintenance. We identified a specific, interdependent expression pattern of activator/repressor transcription factors, miRNAs and miRNA targets with a potential to boost not only epithelial to mesenchymal transition but also chronic inflammation and carcinogenesis in the colon of IBD patients. Notably, there is a clear correlation between expression change of TFs and their regulated miRNAs. Decreased expression of enhancer (e.g. E2F1, HNF1B) and increased expression of repressor (e.g. NFKB1, ZEB2) TFs was associated with the downregulation of all five members of the human miR-200 family (miR-200a, miR-200b, miR-200c, miR-429, and miR-141) and miR-27b. In contrast, elevated miR-223 and miR-31 expression appears to be the consequence of the upregulation of activator (e.g. CEBPB) and decrease of repressor (e.g. E2F1) TFs in the inflamed colon.

miRNAs are potential biomarkers for the diagnosis and prognosis of IBD-associated CRC ( (81) and references within). 21 out of the 68 miRNAs for which we detected altered expression in rat model of colitis are shared by UC-CRC (5 miRNAs), CD-CRC (5 miRNAs) or IBD-CRC (11 miRNAs). Our study revealed altered expression for some of the most studied miRNAs in colitis associated colorectal cancer (e.g. miR-21, -31, -214 and -223), all upregulated which is concordant with previous reports (82). Importantly, miR-31 has been highlighted as a novel biomarker for neoplastic progression in ulcerative colitis (83). The identification of miRNAs as diagnostic biomarkers can revolutionize the screening of IBD patients with the aim to identify those that are at high risk for tumor development.

Abnormal overexpression of the pro-inflammatory cytokine TNFα plays a central role in the development of inflammatory diseases, among others IBD, hence TNFα is a common target of IBD therapies. TNFα is stored as inactive pro-TNFα until it is converted to active form by matrix metalloproteinases, such as MMP13 (70). Both in the inflamed colon samples of TNBS treated rats as well as in IBD patients, elevated expression of TNFα (24) is associated with markedly increased MMP13 expression. The significantly decreased expression of miR-27b, a posttranscriptional regulator of MMP13 (74) may, at least in part, cause MMP13 upregulation, and - indirectly - increase of TNFα.

Colon tissue is composed of over 100 different cell types and their various developmental stages as defined by scRNA-seq (84). These cells differently contribute to the maintenance of mucosal homeostasis or to the regulation of immune response. One of the miRNAs markedly upregulated in inflamed colon is miR-233, a known cargo of microvesicles and exosomes of peripheral blood. Since leukocytes are able to transfer miR-223 containing cargo to alveolar-epithelial cells it is reasonable to propose that, in inflamed colon of IBD patients, myeloid immune cells may exert a similar effect on colonic epithelial cells through miR-233. Our data supports this hypothesis: through the miR-233/E2F1/miR-27b/MMP13 regulatory axis (**Figure 6B**) monocyte-derived miR-233 may result in MMP13 (and possibly other factors’) upregulation (**Figure 8**).

In conclusion, our data together with published findings indicate that changes in microRNAome are associated with the pathophysiology of IBD, consequently, microRNAs are potential targets for the diagnosis, prognosis and treatment of IBD.

## Data availability statement

The data presented in the study are deposited in the NCBI Sequence Read Archive (SRA) database (https://www.ncbi.nlm.nih.gov/) repository, accession number PRJNA837238.

## Ethics statement

The studies involving human participants were reviewed and approved by Hungarian Medical Research Council (ETT) Medical Research Council’s Committee of Scientific and Research Ethics (TUKEB, (license number 59-32-PTE2015/IBD)). The patients/participants provided their written informed consent to participate in this study. The animal study was reviewed and approved by University of Szeged.

## Author contributions

Conceptualization, ÉB and IN. Methodology, ÉB, ZK, ZH, and IN. Validation, ÉB and IN. Formal analysis, ÉB and ZH. Resources, ZK and PB. Data curation, ZH. Writing—original draft preparation, ÉB and IN. Visualization, ÉB and ZH. Supervision, IN. Project administration, ÉB, ZK, and IN. Funding acquisition, IN. All authors contributed to the article and approved the submitted version.

## Funding

This work was funded, in part, by grant from the National Research, Development and Innovation Office (grant number GINOP-2.3.2-15-2016-00039). ÉB was funded by the European Union and the State of Hungary, co-financed by the European Social Fund in the framework of ‘National Excellence Program’ (grant number A2-ELMH-12-0082) and supported by NTP-NFTÖ-19-B (grant number NTP-NFTÖ-19-B-0076).

## Acknowledgments

The authors thank Csaba Varga (Department of Physiology, Anatomy and Neuroscience, University of Szeged) for providing rat samples as well as Patrícia Sarlós and Áron Vincze (1st Department of Internal Medicine, University of Pécs) for obtaining colonic biopsies.

## Conflict of interest

Authors ÉB and IN were employed by Seqomics Biotechnology Ltd.

The remaining authors declare that the research was conducted in the absence of any commercial or financial relationships that could be construed as a potential conflict of interest.

## Publisher’s note

All claims expressed in this article are solely those of the authors and do not necessarily represent those of their affiliated organizations, or those of the publisher, the editors and the reviewers. Any product that may be evaluated in this article, or claim that may be made by its manufacturer, is not guaranteed or endorsed by the publisher.
