# Supplementary material for: Global alteration of colonic microRNAome landscape associated with inflammatory bowel disease
Source: Front Immunol. 2022 Sep 13;13:991346. doi: 10.3389/fimmu.2022.991346 (PMC9513375; doi:10.3389/fimmu.2022.991346)
Supplement: Supplementary file 1 [file DataSheet_1.docx]

Supplementary Material

# Supplementary Figures and Tables

## Supplementary Tables

**Supplementary Table 1.** List of rat miRNAs with significantly altered expression in inflamed versus uninflamed colon samples as determined by miRNA-Seq. ND = No data.

| **Sequence** | **Name** | **Fold change** | **FDR** | **Expression alteration in colonic tissue of IBD patients** |
| --- | --- | --- | --- | --- |
| GAAGTTGTTCGTGGTGGATTCG | mir-382 | -46.76 | 0.02 | Unaltered (Fasseu et al., 2010) |
| ATGACCTATGATTTGACAGACA | mir-215 | -34.47 | 3.00E-25 | Controversial (Peck et al., 2015, Mohammadi et al., 2019) |
| TAATACTGCCTGGTAATGATGAC | mir-200b | -29.25 | 1.26E-31 | Downregulated (Zahm et al., 2014) |
| ACAACAACACCAAACCACCTGA | mir-196c | -27 | 6.37E-07 | Unknown human homolog |
| TAATACTGTCTGGTAATGCCGT | mir-429 | -26.42 | 8.43E-19 | Downregulated (Zidar et al., 2016) |
| TTTGTTCGTTCGGCTCGCGTGA | mir-375 | -24.83 | 0 | Downregulated (Wu et al., 2008) |
| CTGACCTATGAATTGACAGCC | mir-192 | -23.38 | 1.69E-16 | Downregulated (Wu et al., 2008) |
| TAACACTGTCTGGTAACGATGT | mir-200a | -22.28 | 6.99E-17 | Downregulated (Zidar et al., 2016) |
| TCCTTCATTCCACCGGAGTCTGT | mir-205 | -21.43 | 3.02E-22 | ND |
| TAATACTGCCGGGTAATGATG | mir-200c | -19.75 | 4.87E-21 | Downregulated (Zidar et al., 2016) |
| TATGGCACTGGTAGAATTCACT | mir-183 | -17.13 | 1.01E-18 | ND |
| CATCTTACCGGACAGTGCTGG | mir-200a | -16.84 | 1.02E-10 | Downregulated (Zidar et al., 2016) |
| TGTAACAGCAACTCCATGTGGA | mir-194-1//mir-194-2 | -16.2 | 5.81E-10 | Downregulated (Zahm et al., 2014) |
| TAGGTAGTTTCGTGTTGTTGGG | mir-196c | -14.7 | 1.06E-12 | ND |
| GTGAAATGTTTAGGACCACTAG | mir-203a | -14.18 | 2.76E-10 | Downregulated (Mohammadi et al., 2019) |
| CTGCCAGTTCCATAGGTCACAG | mir-192 | -12.07 | 3.58E-08 | Downregulated (Wu et al., 2008) |
| TTTGGCAATGGTAGAACTCACACCG | mir-182 | -10.9 | 7.15E-13 | Upregulated (Xu et al., 2022) |
| TCCATCTTCCAGTGCAGTGTTG | mir-141 | -9.16 | 9.52E-03 | Downregulated (Cai et al., 2017) |
| CCAGTGGGGCTGCTGTTATCT | mir-194-2 | -8.94 | 2.12E-08 | Downregulated (Zahm et al., 2014) |
| CATCTTACTGGGCAGCATTGGA | mir-200b | -8.35 | 3.89E-08 | Downregulated (Zahm et al., 2014) |
| TAGGTAGTTTCATGTTGTTGGG | mir-196a | -8.08 | 3.75E-05 | Upregulated (Fasseu et al., 2010) |
| TAACACTGTCTGGTAAAGATGG | mir-141 | -7.6 | 3.56E-06 | Downregulated (Cai et al., 2017) |
| TAGGTAGTTTCCTGTTGTTGGG | mir-196b-1//mir-196b-2 | -7.39 | 2.29E-10 | Downregulated (Peck et al., 2015) |
| AGCTGGTGTTGTGAATCAGGCCG | mir-138-2//mir-138-1 | -5.67 | 0.01 | Upregulated (Valmiki et al., 2017) |
| TGAGGTTGGTGTACTGTGTGTGA | mir-672 | -4.78 | 0.05 | Unknown human homolog |
| AACATTCATTGTTGTCGGTGGGT | mir-181d | -4.47 | 3.25E-07 | ND |
| ACTGGACTTGGAGTCAGAAGG | mir-378a | -3.73 | 1.77E-07 | Downregulated (Dubois-Camacho et al., 2019) |
| AAAGTTCTGAGACACTCTGACTC | mir-148a | -3.53 | 9.59E-04 | ND |
| AGTGGACTTGGAGTCAGAAGG | mir-378b | -3.52 | 1.85E-05 | Downregulated (Palmieri et al., 2017) |
| AACATTCAACCTGTCGGTGAGT | mir-181c | -3.43 | 3.97E-07 | ND |
| TCGAGGAGCTCACAGTCTAGT | mir-151 | -3.4 | 5.28E-06 | ND |
| AAGGTAGATAGAACAGGTCTTG | mir-1839 | -3.21 | 0.03 | Unknown human homolog |
| TTCACAGTGGCTAAGTTCTGC | mir-27b | -3.16 | 1.90E-06 | ND |
| TGAGGTAGTAGATTGTATAGTT | let-7f-1//let-7f-2 | -3.04 | 3.35E-06 | Upregulated (Wu et al., 2008) |
| AGCAGCATTGTACAGGGCTATCA | mir-107 | -2.95 | 6.65E-04 | ND |
| ACCATCGACCGTTGAGTGGACC | mir-181c | -2.65 | 0.01 | ND |
| CAGCAGCACACTGTGGTTTGTA | mir-497 | -2.49 | 2.77E-03 | ND |
| CAACGGAATCCCAAAAGCAGCTG | mir-191a | -2.47 | 1.56E-04 | Upregulated (Wu et al., 2010) |
| CTTTCAGTCGGATGTTTGCAGC | mir-30a | -2.43 | 3.79E-03 | ND |
| AAGGGATTCTGATGTTGGTCACACT | mir-541 | -2.41 | 0.04 | ND |
| ACAGTAGTCTGCACATTGGTTA | mir-199a | -2.4 | 1.01E-03 | ND |
| TGTAAACATCCTCGACTGGAAG | mir-30a | -2.34 | 6.65E-04 | ND |
| CCCTGTAGAACCGAATTTGTGT | mir-10b | -2.25 | 7.16E-03 | Downregulated (Van der Goten et al., 2014) |
| TTCAAGTAATCCAGGATAGGCT | mir-26a | -2.15 | 2.68E-03 | Upregulated (Fasseu et al., 2010) |
| TAGCTTATCAGACTGATGTTGA | mir-21 | 2.03 | 0.01 | Upregulated (Wu et al., 2008) |
| TAACAGTCTACAGCCATGGTCG | mir-132 | 2.13 | 0.01 | ND |
| CCATGGATCTCCAGGTGGGT | mir-490 | 2.26 | 0.04 | ND |
| AGGCAAGATGCTGGCATAGCTG | mir-31a | 2.4 | 0.05 | Upregulated (Van der Goten et al., 2014) |
| ACAGCAGGCACAGACAGGCAG | mir-214 | 2.46 | 0.01 | Upregulated (Polytarchou et al., 2015) |
| ACCATCGACCGTTGATTGTACC | mir-181a-1 | 2.49 | 3.38E-03 | ND |
| TGCCCACCCTTTACCCCACTCCA | mir-702 | 2.55 | 3.20E-03 | Unknown human homolog |
| TCTAGGGCTGGAGAGATGGCTA | mir-3473 | 3.45 | 4.98E-05 | Unknown human homolog |
| TAACAGTCTCCAGTCACGGCCA | mir-212 | 3.68 | 1.19E-04 | ND |
| AGGCCTGCTCTGAGCCCCCGC | mir-6328 | 3.99 | 5.81E-06 | Unknown human homolog |
| ACGGGTTAGGCTCTTGGGAGCT | mir-125b-1 | 4.03 | 1.28E-06 | Upregulated (Valmiki et al., 2017) |
| TCCTGTACTGAGCTGCCCCGAG | mir-486 | 4.34 | 1.00E-06 | ND |
| CATTATTACTTTTGGTACGCG | mir-126a | 4.37 | 1.34E-05 | Upregulated (Wu et al., 2008) |
| TCCCTGAGGAGCCCTTTGAGCCTGA | mir-351-1//mir-351-2 | 4.78 | 3.56E-08 | Unknown human homolog |
| AGCCACTGCCCACAGCACACTG | mir-210 | 4.82 | 1.87E-03 | Upregulated (Bakirtzi et al., 2016) |
| ACCTTGGCTCTAGACTGCTTACTG | mir-212 | 5 | 3.17E-03 | ND |
| TTATAAAGCAATGAGACTGATT | mir-340-1//mir-340-2 | 6.65 | 1.85E-05 | ND |
| CATAAAGTAGAAAGCACTACT | mir-142 | 9.03 | 4.74E-07 | Controversial (Zahm et al., 2014) |
| GATACACAGAGGCAGGAGGAGAA | mir-6216 | 10.12 | 3.56E-06 | Unknown human homolog |
| CTCGGGGATCATCATGTCACGA | mir-542-1//mir-542-2//mir-542-3 | 10.94 | 0.03 | ND |
| GGCAGAGGAGGGCTGTTCTTCCC | mir-298 | 12.45 | 1.59E-09 | ND |
| TGTCAGTTTGTCAAATACCCC | mir-223 | 14.4 | 1.36E-19 | Upregulated (Valmiki et al., 2017) |
| TGGCAGTGTATTGTTAGCTGGT | mir-449a | 35.44 | 1.18E-05 | Upregulated (Feng et al., 2018) |
| AAACCGTTACCATTACTGAGTT | mir-451 | 37.51 | 7.31E-04 | Upregulated (Wu et al., 2017) |

BAKIRTZI, K., LAW, I. K., XUE, X., ILIOPOULOS, D., SHAH, Y. M. & POTHOULAKIS, C. 2016. Neurotensin Promotes the Development of Colitis and Intestinal Angiogenesis via Hif-1alpha-miR-210 Signaling. *J Immunol,* 196**,** 4311-21.

CAI, M., CHEN, S. & HU, W. 2017. MicroRNA-141 Is Involved in Ulcerative Colitis Pathogenesis via Aiming at CXCL5. *J Interferon Cytokine Res,* 37**,** 415-420.

DUBOIS-CAMACHO, K., DIAZ-JIMENEZ, D., DE LA FUENTE, M., QUERA, R., SIMIAN, D., MARTINEZ, M., LANDSKRON, G., OLIVARES-MORALES, M., CIDLOWSKI, J. A., XU, X., GAO, G., XIE, J., CHNAIDERMAN, J., SOTO-RIFO, R., GONZALEZ, M. J., CALIXTO, A. & HERMOSO, M. A. 2019. Inhibition of miR-378a-3p by Inflammation Enhances IL-33 Levels: A Novel Mechanism of Alarmin Modulation in Ulcerative Colitis. *Front Immunol,* 10**,** 2449.

FASSEU, M., TRETON, X., GUICHARD, C., PEDRUZZI, E., CAZALS-HATEM, D., RICHARD, C., APARICIO, T., DANIEL, F., SOULE, J. C., MOREAU, R., BOUHNIK, Y., LABURTHE, M., GROYER, A. & OGIER-DENIS, E. 2010. Identification of restricted subsets of mature microRNA abnormally expressed in inactive colonic mucosa of patients with inflammatory bowel disease. *PLoS One,* 5.

FENG, Y., DONG, Y. W., SONG, Y. N., XIAO, J. H., GUO, X. Y., JIANG, W. L. & LU, L. G. 2018. MicroRNA449a is a potential predictor of colitisassociated colorectal cancer progression. *Oncol Rep,* 40**,** 1684-1694.

MOHAMMADI, A., KELLY, O. B., SMITH, M. I., KABAKCHIEV, B. & SILVERBERG, M. S. 2019. Differential miRNA Expression in Ileal and Colonic Tissues Reveals an Altered Immunoregulatory Molecular Profile in Individuals With Crohn's Disease versus Healthy Subjects. *J Crohns Colitis,* 13**,** 1459-1469.

PALMIERI, O., CREANZA, T. M., BOSSA, F., LATIANO, T., CORRITORE, G., PALUMBO, O., MARTINO, G., BISCAGLIA, G., SCIMECA, D., CARELLA, M., ANCONA, N., ANDRIULLI, A. & LATIANO, A. 2017. Functional Implications of MicroRNAs in Crohn's Disease Revealed by Integrating MicroRNA and Messenger RNA Expression Profiling. *Int J Mol Sci,* 18.

PECK, B. C., WEISER, M., LEE, S. E., GIPSON, G. R., IYER, V. B., SARTOR, R. B., HERFARTH, H. H., LONG, M. D., HANSEN, J. J., ISAACS, K. L., TREMBATH, D. G., RAHBAR, R., SADIQ, T. S., FUREY, T. S., SETHUPATHY, P. & SHEIKH, S. Z. 2015. MicroRNAs Classify Different Disease Behavior Phenotypes of Crohn's Disease and May Have Prognostic Utility. *Inflamm Bowel Dis,* 21**,** 2178-87.

POLYTARCHOU, C., HOMMES, D. W., PALUMBO, T., HATZIAPOSTOLOU, M., KOUTSIOUMPA, M., KOUKOS, G., VAN DER MEULEN-DE JONG, A. E., OIKONOMOPOULOS, A., VAN DEEN, W. K., VORVIS, C., SEREBRENNIKOVA, O. B., BIRLI, E., CHOI, J., CHANG, L., ANTON, P. A., TSICHLIS, P. N., POTHOULAKIS, C., VERSPAGET, H. W. & ILIOPOULOS, D. 2015. MicroRNA214 Is Associated With Progression of Ulcerative Colitis, and Inhibition Reduces Development of Colitis and Colitis-Associated Cancer in Mice. *Gastroenterology,* 149**,** 981-92 e11.

VALMIKI, S., AHUJA, V. & PAUL, J. 2017. MicroRNA exhibit altered expression in the inflamed colonic mucosa of ulcerative colitis patients. *World J Gastroenterol,* 23**,** 5324-5332.

VAN DER GOTEN, J., VANHOVE, W., LEMAIRE, K., VAN LOMMEL, L., MACHIELS, K., WOLLANTS, W. J., DE PRETER, V., DE HERTOGH, G., FERRANTE, M., VAN ASSCHE, G., RUTGEERTS, P., SCHUIT, F., VERMEIRE, S. & ARIJS, I. 2014. Integrated miRNA and mRNA expression profiling in inflamed colon of patients with ulcerative colitis. *PLoS One,* 9**,** e116117.

WU, F., ZHANG, S., DASSOPOULOS, T., HARRIS, M. L., BAYLESS, T. M., MELTZER, S. J., BRANT, S. R. & KWON, J. H. 2010. Identification of microRNAs associated with ileal and colonic Crohn's disease. *Inflamm Bowel Dis,* 16**,** 1729-38.

WU, F., ZIKUSOKA, M., TRINDADE, A., DASSOPOULOS, T., HARRIS, M. L., BAYLESS, T. M., BRANT, S. R., CHAKRAVARTI, S. & KWON, J. H. 2008. MicroRNAs are differentially expressed in ulcerative colitis and alter expression of macrophage inflammatory peptide-2 alpha. *Gastroenterology,* 135**,** 1624-1635 e24.

WU, L. Y., MA, X. P., SHI, Y., BAO, C. H., JIN, X. M., LU, Y., ZHAO, J. M., ZHOU, C. L., CHEN, D. & LIU, H. R. 2017. Alterations in microRNA expression profiles in inflamed and noninflamed ascending colon mucosae of patients with active Crohn's disease. *J Gastroenterol Hepatol,* 32**,** 1706-1715.

XU, Y., YANG, J., CHEN, X., DENG, J., GONG, H., LI, F. & OUYANG, M. 2022. MicroRNA-182-5p aggravates ulcerative colitis by inactivating the Wnt/beta-catenin signaling pathway through DNMT3A-mediated SMARCA5 methylation. *Genomics,* 114**,** 110360.

ZAHM, A. M., HAND, N. J., TSOUCAS, D. M., LE GUEN, C. L., BALDASSANO, R. N. & FRIEDMAN, J. R. 2014. Rectal microRNAs are perturbed in pediatric inflammatory bowel disease of the colon. *J Crohns Colitis,* 8**,** 1108-17.

ZIDAR, N., BOSTJANCIC, E., JERALA, M., KOJC, N., DROBNE, D., STABUC, B. & GLAVAC, D. 2016. Down-regulation of microRNAs of the miR-200 family and up-regulation of Snail and Slug in inflammatory bowel diseases - hallmark of epithelial-mesenchymal transition. *J Cell Mol Med,* 20**,** 1813-20.
